# Additional Diagnostic Yield of Endocervical Curettage in Type 3 Transformation Zone for High-Grade Cervical Lesions: A Retrospective Analysis by Human Papillomavirus Genotype

**DOI:** 10.3390/diagnostics16081220

**Published:** 2026-04-19

**Authors:** Elena Lavinia Rusu, Victor Bogdan Buciu, Lavinia Balan, Denis Mihai Serban, Jasmina Chiriac, Veronica Daniela Chiriac

**Affiliations:** 1 Doctoral School, “Victor Babes” University of Medicine and Pharmacy, E. Murgu Square, No. 2, 300041 Timisoara, Romania; elena.rusu@umft.ro (E.L.R.); chiriac_jasmina98@yahoo.com (J.C.); 2 Department of Obstetrics and Gynecology, “Victor Babes” University of Medicine and Pharmacy, Eftimie Murgu Square, No. 2, 300041 Timisoara, Romania; lavinia.balan@umft.ro (L.B.); denis.serban@umft.ro (D.M.S.); chiriac.veronica@umft.ro (V.D.C.)

**Keywords:** colposcopy, endocervical curettage, transformation zone 3, cervical intraepithelial neoplasia, additional yield, biopsy

## Abstract

**Background/Objectives:** When the squamocolumnar junction is not fully visible (type 3 transformation zone), colposcopy-directed biopsy may under-sample endocervical disease. Endocervical curettage (ECC) is recommended in selected settings, but its incremental diagnostic yield in this setting, and whether this yield is concentrated in women with HPV16/18, remains clinically debated. **Methods:** We performed a retrospective cohort study of women referred to colposcopy because of HPV16/18 positivity regardless of cytology, persistent non-16/18 high-risk HPV positivity, and non-16/18 high-risk HPV positivity with abnormal cytology. Persistent non-16/18 high-risk HPV positivity was defined as repeated positivity on two tests performed at least 6 months apart. Eligible women had type 3 transformation zone documented and underwent paired ectocervical biopsy plus ECC at the same visit; biopsy was obtained in all women, including targeted sampling of the most abnormal ectocervical area when no discrete lesion was evident. Women were stratified by HPV genotype into HPV16/18 and non-16/18 high-risk HPV groups. The primary outcome was index high-grade cervical lesion, defined histologically as CIN2, CIN3, or carcinoma in situ; invasive cervical cancer was excluded. The added diagnostic yield of ECC was defined as ECC-only CIN2+, that is, CIN2+ detected on ECC when biopsy was <CIN2. **Results:** The cohort included 690 women (HPV16/18: 310; non-16/18 high-risk HPV: 380). Baseline cytology was negative for intraepithelial lesion or malignancy in 116 individuals (16.8%), ASC-US in 155 (22.5%), LSIL in 205 (29.7%), and ASC-H/HSIL in 214 (31.0%). On index composite histology, 122/690 (17.7%) had CIN2, 198/690 (28.7%) had CIN3, and 11/690 (1.6%) had carcinoma in situ. ECC identified CIN2+ not detected by biopsy in 19/690 (2.8%) cases, representing 19/331 (5.7%) of all CIN2+ diagnoses. ECC-only CIN2+ was more frequent in HPV16/18 than non-16/18 high-risk HPV (4.5% vs. 1.3%, *p* = 0.017). In multivariable analysis, HPV16/18 was associated with increased odds of ECC-only CIN2+ (aOR 3.22, 95% CI 1.10–9.44). No invasive cervical cancers were included in the analytic cohort. **Conclusions:** In type 3 transformation zone colposcopy, ECC adds a modest incremental yield for CIN2+ detection, with higher yield in HPV16/18-positive women. These findings support a risk-weighted approach to ECC rather than universal routine sampling.

## 1. Introduction

Cervical cancer remains a major and largely preventable cause of cancer morbidity and mortality worldwide. Contemporary global estimates based on GLOBOCAN 2022 data confirm a substantial annual burden, with marked geographic inequalities that closely track differences in HPV vaccination coverage, screening access, and timely treatment of precancerous lesions [1,2,3]. The WHO elimination strategy has further emphasized that high-performance screening and effective management of cervical precancer are central levers for reducing incidence and mortality at population level [1,4].

High-risk human papillomavirus (hrHPV) infection is the necessary causal factor for virtually all cervical cancers, and screening is increasingly centered on HPV-based testing with risk stratification to guide colposcopy and treatment [5,6,7]. Within hrHPV types, HPV16 and HPV18 consistently represent the highest-oncogenic-potential group, and genotype information is increasingly used to triage women toward colposcopy and closer surveillance [7,8]. Large longitudinal screening datasets and risk-stratification studies also show that HPV16/18 positivity is associated with the highest immediate and cumulative risk for CIN3+ compared with other genotypes, supporting genotype-informed management thresholds in modern guidance [6,9].

Colposcopy remains the key diagnostic step after abnormal screening, aiming to detect clinically relevant high-grade disease and obtain representative histology for management [10,11]. Even with contemporary standards, colposcopy is imperfect, and sensitivity improves when clinicians obtain more than one targeted biopsy in the presence of multiple acetowhite areas [10,12]. A persistent challenge arises when the squamocolumnar junction is not fully visible, classified as a type 3 transformation zone (TZ3), because clinically relevant lesions may extend into the endocervical canal beyond direct visualization [11,13,14]. This anatomical limitation is particularly relevant in older women and in those with risk factors for endocervical disease, where visual impression and ectocervical biopsy alone may under-sample the true extent of precancer [7,13].

Endocervical curettage (ECC) was developed to address this blind spot by sampling the endocervical canal during colposcopy [10,14]. Updated colposcopy standards and consensus recommendations specifically support ECC when the squamocolumnar junction is not fully visualized (TZ3) and in higher-risk referral profiles, including high-grade cytology and HPV16/18 infection; ECC is also preferred in older age groups in several guidance documents [14]. At the same time, ECC is not a benign “add-on.” It can increase procedure time and discomfort, and its overall incremental diagnostic yield is often modest, which explains the ongoing debate about when ECC meaningfully changes management. Recent clinical studies report incremental ECC yields for CIN2+ that are generally in the low single digits, while also suggesting added yield concentrates in selected subgroups such as high-grade cytology, HPV16/18 positivity, older or postmenopausal patients, and those with TZ3 anatomy [15,16,17].

Despite clear biologic plausibility, clinically useful evidence is still heterogeneous regarding the extent to which ECC adds actionable detection specifically in TZ3 colposcopy, and whether this incremental yield is disproportionately driven by HPV16/18 infections compared with non-16/18 hrHPV [14,15]. Clarifying this point matters because HPV16/18 status is already a central risk modifier in contemporary cervical screening pathways, and a more selective ECC strategy could potentially preserve detection while reducing unnecessary instrumentation in lower-yield scenarios [6,18,19]. Therefore, in a retrospective cohort of women with TZ3 colposcopy undergoing paired biopsy and ECC, we evaluated the added diagnostic yield of ECC for detecting histologic CIN2+ and examined whether ECC-only CIN2+ detection is concentrated among women with HPV16/18 compared with those with non-16/18 hrHPV [16,20,21].

## 2. Methods

### 2.1. Study Design and Setting

We conducted a retrospective observational cohort study in a tertiary university-affiliated center of Obstetrics and Gynaecology in western Romania. The study period extended from 1 January 2022 to 31 December 2025. The source population consisted of adult women referred to colposcopy within routine clinical care pathways. Data were retrieved from institutional electronic records and pathology archives, and were abstracted into a dedicated, de-identified research dataset. The study was designed and reported in line with STROBE principles for observational studies.

The index visit was defined as the colposcopy encounter at which the transformation zone was classified as type 3 (TZ3) and paired tissue sampling was performed.

### 2.2. Exposure Definition

The primary exposure was HPV genotype risk group, categorized as HPV16/18 versus non-16/18 high-risk HPV (hrHPV) based on available genotyping results recorded before or at the index colposcopy visit. For descriptive purposes, the HPV16/18 group included HPV16 and/or HPV18 positivity, while the non-16/18 group included hrHPV positivity without HPV16 or HPV18.

### 2.3. Inclusion and Exclusion Criteria

Eligible women met all of the following criteria: age ≥18 years; referral for colposcopy between 1 January 2022 and 31 December 2025 for HPV16/18 positivity regardless of cytology, persistent non-16/18 high-risk human papillomavirus positivity, or non-16/18 high-risk human papillomavirus positivity with abnormal cytology; type 3 transformation zone at the index colposcopy; available HPV genotyping allowing classification as HPV16/18 or non-16/18 high-risk human papillomavirus; paired ectocervical biopsy and endocervical curettage performed at the same visit; and interpretable index histology.

Exclusion criteria were: absence of paired ectocervical biopsy and endocervical curettage at the same visit; missing HPV genotyping; insufficient documentation to assign an index histologic outcome; invasive cervical carcinoma diagnosed before inclusion or on index histology; prior gynecologic oncologic disease; and prior surgical intervention involving the cervix, vagina, or uterus that could alter colposcopic or histologic assessment.

### 2.4. Referral Indications and Operational Definitions

Women were referred for colposcopy if at least one of the following applied: (1) HPV16 and/or HPV18 positivity regardless of cytology; (2) persistent non-16/18 high-risk human papillomavirus positivity, defined as repeated non-16/18 high-risk human papillomavirus positivity on two tests at least 6 months apart; or (3) non-16/18 high-risk human papillomavirus positivity with abnormal cytology. Abnormal cytology was defined as LSIL or worse.

### 2.5. Colposcopy and Sampling Procedures

Colposcopy was performed according to local regulations. The transformation zone was classified by the examining clinician as TZ3 when the squamocolumnar junction was not fully visible. Endocervical curettage was performed during the same encounter using routine technique to sample the endocervical canal. ECC specimens were processed and reported by the pathology department as diagnostic categories or, when appropriate, as insufficient for diagnosis when the specimen contained inadequate evaluable endocervical epithelium (for example, scant tissue, blood/mucus only, or artifact precluding interpretation). In this study, insufficient ECC was retained as a specimen adequacy descriptor and was not treated as a negative result.

### 2.6. Histopathology and Outcome Definitions

All histology results were extracted from pathology reports. The primary endpoint was index composite CIN2+, defined as cervical intraepithelial neoplasia grade 2 or worse, namely CIN2, CIN3, or carcinoma in situ (CIS). The index composite histologic outcome for each participant was defined as the highest-grade diagnosis identified on either directed biopsy or ECC at the index visit. For descriptive reporting, index composite outcomes were summarized as no dysplasia/CIN1, CIN2, CIN3, and CIS. Subsequent conization, when performed within 12 weeks on routine clinical grounds, was recorded descriptively to contextualize discordant or insufficient index histology.

Women with index histology below CIN2 were managed according to routine clinical judgment based on referral risk, cytology, colposcopic impression, transformation zone type, and specimen adequacy. Immediate conization was considered in selected cases with persistent high-grade discordance or insufficient endocervical sampling despite persistent high-risk referral features, whereas women not selected for immediate excision underwent conservative follow-up with repeat HPV testing, cytology, and/or repeat colposcopy according to local protocol. For this study, persistent high-grade discordance was defined as high-grade referral cytology (ASC-H/HSIL) in the setting of a type 3 transformation zone with index biopsy and ECC below CIN2. Persistent high-risk referral features were defined as HPV16/18 positivity persisting for at least 6 months, persistent non-16/18 high-risk human papillomavirus positivity persisting for at least 6 months, ASC-H or HSIL cytology, or high-grade colposcopic impression despite non-diagnostic or insufficient ECC. No invasive cervical cancers were included in the analytic cohort. 

To assess the incremental diagnostic value of ECC beyond biopsy, high-grade lesion cases were classified into three mutually exclusive groups: (1) biopsy-only cases, defined as CIN2 or worse detected by biopsy with ECC below CIN2 or non-diagnostic; (2) concordant cases, defined as CIN2 or worse detected by both biopsy and ECC; and (3) ECC-only cases, defined as CIN2 or worse detected by ECC with biopsy below CIN2. Cases in the ECC-only group were considered to represent the additional diagnostic yield of ECC.

### 2.7. Statistical Analysis

Data entry and cleaning were performed using Microsoft Excel 2016 (Microsoft Corp., Redmond, WA, USA) [22]. Statistical analyses were conducted using IBM SPSS Statistics version 26 (IBM Corp., Armonk, NY, USA) [23] and R version 4.3.3 (R Foundation for Statistical Computing, Vienna, Austria) [24].

Continuous variables were assessed for distributional assumptions and are presented as mean ± standard deviation when approximately symmetric. Categorical variables are presented as counts and percentages. Between-group comparisons (HPV16/18 vs. non-16/18 hrHPV) were performed using Welch’s *t*-test for continuous variables and chi-square testing for categorical variables. Fisher’s exact test was used when expected cell counts were small, particularly for ECC-only CIN2+.

The incremental yield of ECC was reported as the proportion of ECC-only CIN2+ cases in the full cohort and within HPV strata. Confidence intervals for incremental yield were estimated using Wilson methods for proportions. The number needed for ECC to detect one additional CIN2+ case was calculated as the reciprocal of the ECC-only CIN2+ proportion.

A multivariable logistic regression model was constructed with ECC-only CIN2+ as the dependent outcome to evaluate independent associations with HPV16/18 status while adjusting for prespecified clinical covariates (age ≥40 years, high-grade cytology, and high-grade colposcopic impression). Effect sizes are reported as adjusted odds ratios (aORs) with 95% confidence intervals. All tests were two-sided, and *p*-values < 0.05 were considered statistically significant.

### 2.8. Ethical Considerations

The study was conducted in accordance with the Declaration of Helsinki [25] and applicable data protection requirements. Given the retrospective, non-interventional design and the use of anonymized routinely collected clinical data, the requirement for individual informed consent was waived according to institutional policy. The protocol was approved by the local Ethics Committee (approval number 117/1 January 2022). Data handling complied with the General Data Protection Regulation (EU 2016/679) [26].

## 3. Results

### 3.1. Study Population

Between January 2022 and December 2025, 690 women met the study eligibility criteria and were included in the analytic cohort. Of these, 310/690 (44.9%) were classified in the HPV16/18 group and 380/690 (55.1%) in the non-16/18 high-risk human papillomavirus group. An insufficient ECC specimen was recorded in 158/690 (22.9%). Baseline characteristics are summarized in Table 1.

### 3.2. Baseline Characteristics

Patients in the HPV16/18 group were slightly younger than those with non-16/18 hrHPV (mean 38.9 ± 8.8 vs. 40.6 ± 9.8 years; *p* = 0.019). The proportion aged ≥40 years was lower in HPV16/18 (39.0%) than non-16/18 (49.2%; *p* = 0.009).

High-grade cytology (ASC-H/HSIL) was more frequent in HPV16/18 (42.6%) than in non-16/18 (21.6%; *p* < 0.001). A high-grade colposcopic impression was also more frequent in HPV16/18 (35.5%) than non-16/18 (18.9%; *p* < 0.001). The rate of insufficient ECC was similar between groups (*p* = 0.414). Details are shown in Table 1.

### 3.3. Histologic Outcomes and Detection Pathways

On index composite histology, defined as the highest-grade diagnosis on paired biopsy and/or ECC at the index visit, 331/690 (48.0%) women had CIN2+ and 209/690 (30.3%) had CIN3+. Index composite outcomes were no dysplasia/CIN1 in 359 (52.0%), CIN2 in 122 (17.7%), CIN3 in 198 (28.7%), and CIS in 11 (1.6%). No invasive cervical cancers were included in the analytic cohort.

CIN2+ was more common in HPV16/18 than non-16/18 hrHPV (60.6% vs. 37.6%, *p* < 0.001), and the same pattern was observed for CIN3+ (41.3% vs. 21.3%, *p* < 0.001).

### 3.4. Index Composite Histology and Added Diagnostic Yield of ECC

Across the full cohort, 19/690 (2.8%) cases of index composite CIN2+ were detected only by ECC, representing the incremental yield of ECC beyond ectocervical biopsy. In absolute terms, this corresponds to 19/331 (5.7%) of all CIN2+ diagnoses in the cohort with ECC-only.

**Figure 1:**
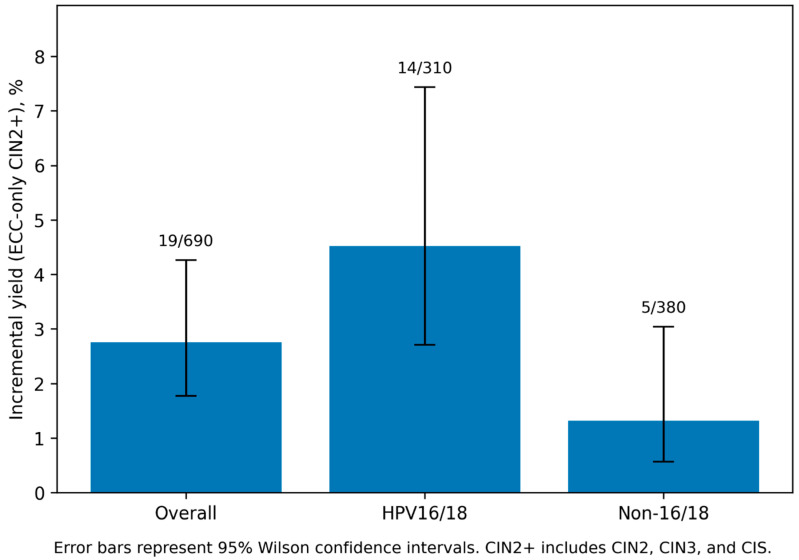
Incremental yield of ECC (ECC-only CIN2+) by HPV group. Overall: 2.8% (19/690), 95% CI 1.8–4.3%, HPV16/18: 4.5% (14/310), 95% CI 2.7–7.4%; Non-16/18 hrHPV: 1.3% (5/380), 95% CI 0.6–3.0%; Between-group comparison (HPV16/18 vs. non-16/18): *p* = 0.017; 95% CIs are Wilson intervals for proportions. *p*-value from Fisher’s exact test comparing ECC-only CIN2+ rates between HPV groups.

The incremental yield differed by HPV group. ECC-only CIN2+ occurred in 14/310 (4.5%) in HPV16/18 versus 5/380 (1.3%) in non-16/18 hrHPV (*p* = 0.017). The corresponding number needed for ECC to detect one additional CIN2+ was 22.1 in HPV16/18 and 76.0 in non-16/18 hrHPV (overall: 36.3). These results are detailed in Table 2 and visualized in Figure 1.

### 3.5. Subsequent Conization and Clinically Discordant Cases

Subsequent conization within 12 weeks was performed in 268/690 (38.8%) women, more often in the HPV16/18 group than in the non-16/18 high-risk human papillomavirus group (172/310 [55.5%] vs. 96/380 [25.3%], *p* < 0.001). Among these, 214/268 (79.9%) underwent conization because CIN2 or worse had already been identified on index biopsy and/or ECC. The remaining 54/268 (20.1%) underwent conization despite index biopsy and ECC below CIN2. In this subgroup, indications for excision were persistent high-grade discordance in a type 3 transformation zone (37/54, 68.5%) and insufficient ECC with persistent high-risk referral features, as described above (17/54, 31.5%).

Among the 54 women with index biopsy and ECC below CIN2 who underwent conization, 17/54 (31.5%) were upgraded on excisional histology, including 6/54 (11.1%) with CIN2, 10/54 (18.5%) with CIN3, and 1/54 (1.9%) with carcinoma in situ, whereas 37/54 (68.5%) remained below CIN2. Of these 54 women, 33 were HPV16/18-positive and 21 were non-16/18 high-risk human papillomavirus-positive.

Among the 359 women with index histology below CIN2, 305/359 (85.0%) did not undergo conization and were managed conservatively with cytological, genotype and colposcopy reevaluation at 6–12 months. Management in this non-excisional subgroup was similar between HPV genotype groups.

These findings are summarized in Table 3.

## 4. Discussion

### 4.1. Interpretation of the Results

In this TZ3 cohort, ECC provided a modest but clinically relevant diagnostic gain beyond directed biopsy. The overall ECC-only CIN2+ rate was 2.8%, which is consistent with the low single-digit added yield reported for ECC as an adjunctive test rather than a substitute for biopsy [14,16]. This benefit was not evenly distributed. ECC-only CIN2+ was more frequent in HPV16/18-positive women than in those with non-16/18 hrHPV (4.5% vs 1.3%), and HPV16/18 remained independently associated with ECC-only CIN2+ in multivariable analysis. This is clinically plausible, as HPV16/18 is linked to higher short-term risk of high-grade disease, and current guidance gives greater weight to ECC when HPV16/18 is present and the squamocolumnar junction is not fully visible, as in TZ3 [17].

The number needed for ECC reinforces the same point. In our cohort, one additional CIN2+ case was identified for about every 22 ECCs in HPV16/18-positive women, compared with about 76 ECCs in the non-16/18 group. Taken together, these data support a selective, risk-based approach to ECC in TZ3 rather than routine universal use. Most CIN2+ lesions were still detected by biopsy, meaning that ECC was more often complementary than decisive, which is expected when directed sampling is carefully performed.

An additional practical finding was the 22.9% rate of non-diagnostic ECC. The value of ECC depends not only on incremental yield, but also on how often the specimen is interpretable in routine care. This is why we retained “insufficient ECC” as a feasibility marker rather than classifying it as negative histology. Current standards also acknowledge that ECC adequacy depends on sampling technique, handling, and patient tolerance [5,14,27]. Baseline differences in our cohort, including more high-grade cytology and high-grade colposcopic impression in HPV16/18-positive women, further support the interpretation that this group was risk-enriched. More broadly, age and TZ type are known to influence colposcopic performance, which helps explain why TZ3 anatomy and older age are repeatedly emphasized in ECC guidance [28,29].

### 4.2. Comparison with Current Literature

Our overall ECC-only CIN2+ rate of 2.8% is close to the range reported in recent prospective and large retrospective studies. Jareemit et al. reported an added yield of 2.9% for CIN2+ missed by colposcopy-directed biopsy, while a nationwide screening-based analysis also found a low overall additional yield that increased in selected clinical settings [16,17]. In a very large cohort of 47,134 women, the overall added yield of ECC was 1.4%, with greater benefit in those with more severe final disease [30]. Differences across studies likely reflect variation in referral patterns, biopsy strategy, and outcome definitions. In a multicenter TZ3 cohort, ECC identified 0.8% of HSIL+ lesions missed by biopsy alone, which is lower than in our study but still supports the same overall principle: ECC is usually low-yield in unselected populations, but becomes more useful in anatomically or clinically higher-risk groups, including TZ3 [14,31,32,33].

Our finding that the added yield was concentrated in HPV16/18-positive women also agrees with recent evidence. HPV16, or HPV16/18 more broadly, has been identified as a predictor of ECC benefit in both prospective and retrospective series [17,30,31]. Likewise, the association we observed between higher-risk referral features and ECC performance is consistent with the broader literature showing that high-grade cytology and high-grade colposcopic impression increase the likelihood of detecting CIN2+ on ECC [8,9,21,32]. This fits with ASCCP standards, which emphasize ECC particularly when colposcopy is limited by incomplete visualization and when other high-risk factors are present [7]. Overall, the literature supports a simple hierarchy: ECC contributes most when pretest risk is high and the transformation zone is not fully visible.

### 4.3. Type 3 Transformation Zone and the Diagnostic Excision Question

TZ3 should be understood as a limitation of colposcopic visualization rather than a reassuring finding, because clinically relevant disease may extend into the endocervical canal beyond direct view. This is the central reason why ECC is recommended in TZ3 examinations [14,16]. However, ECC does not completely eliminate this blind spot. When screening risk remains high but biopsy and ECC are negative, low-grade, or non-diagnostic, diagnostic excision may still be justified [34,35,36,37]. The 2023 Canadian Colposcopy Guideline supports excision in cases of persistent discordance, particularly when high-grade cytology coexists with TZ3, and European expert guidance similarly recognizes type 3 excision as an appropriate strategy in selected complex cases [38,39]. This is supported by evidence showing that biopsy can underestimate CIN2+ in older women with TZ3 when compared with excisional histology [40]. At the same time, excision is not benign, especially in women with future reproductive plans, since cervical excision is associated with a higher risk of preterm birth [41]. For that reason, escalation should remain selective and risk-based.

This interpretation is also supported by our clinically selected conization subgroup, in which 17 women with biopsy and ECC both <CIN2 at the index visit were subsequently upgraded to CIN2+ on excision, underscoring that negative or low-grade index histology does not fully exclude clinically relevant endocervical disease in TZ3.

An important finding of the clinically selected conization subgroup was that 17/54 women (31.5%) with biopsy and ECC both below CIN2 at the index visit were subsequently upgraded on excisional histology. This proportion should not be interpreted as the false-negative rate of biopsy and ECC in the whole cohort, because these women were selected for excision precisely because persistent high-grade discordance or insufficient endocervical sampling maintained a high level of concern. In type 3 transformation zone examinations, persistent discordance between referral risk and index histology justifies a lower threshold for excision, particularly when high-grade cytology is present [38].

### 4.4. Future Perspectives

Future refinement of TZ3 management will likely depend on better risk stratification. Extended HPV genotyping is already being incorporated into guideline frameworks because not all high-risk genotypes carry the same CIN3+ risk, and this may help identify which TZ3 patients are most likely to benefit from endocervical assessment [42,43]. Biomarker triage is also becoming more relevant. p16/Ki-67 dual stain is now accepted in formal guidance as a triage tool for HPV-positive women, and WHO has also endorsed its use in this setting [44,45]. Methylation-based triage is another promising direction, particularly for non-16/18 infections where management is often less straightforward [46,47,48].

AI-assisted colposcopy is another evolving area. Recent studies suggest that AI-based assessment of cervical images can achieve clinically relevant accuracy for CIN2+/CIN3+ and may reduce observer variability [49,50,51]. Some multicenter studies have already shown that AI can be incorporated into digital image workflows, although heterogeneity in endpoints, devices, and validation remains substantial [52,53,54].

Another useful next step would be a prospective TZ3-focused study with standardized biopsy number, ECC technique, and pathology reporting, along with prespecified criteria for when discordance should prompt excision. Such a design would better define when ECC truly changes management and when newer risk-stratification tools might safely reduce unnecessary procedures.

### 4.5. Limitations

This study has the typical limitations of retrospective colposcopy research. Referral and sampling decisions were made in routine care, so selection bias is possible. ECC adequacy and colposcopic assessment are operator-dependent, and the relatively high rate of insufficient ECC reflects real-world variability in technique and specimen quality [7,37]. In addition, ECC-only CIN2+ was an uncommon endpoint, which limits model stability and increases uncertainty around adjusted estimates. Finally, because TZ3 implies incomplete visualization, verification bias cannot be fully excluded without systematic excisional reference histology. Existing data suggest that biopsy may underestimate CIN2+ in TZ3, especially in older women, so some residual uncertainty remains even after biopsy with or without ECC [40].

Because the cohort was restricted to women who underwent paired ectocervical biopsy and ECC at the same visit, the study may have excluded selected cases in which ECC was performed without biopsy, such as women with a normal-appearing ectocervix but persistent endocervical risk; this may have led to underestimation of the diagnostic contribution of ECC.

Because only a selected subset of women with index histology below CIN2 underwent conization, upgrade rates in this subgroup reflect clinician-selected high-risk cases and should not be generalized to all women with negative or low-grade index histology.

## 5. Conclusions

In women with TZ3, ECC provided a small but clinically meaningful added yield for detecting CIN2+ beyond directed biopsy. This benefit was concentrated in HPV16/18-positive women, supporting a selective rather than universal approach to ECC in this setting. TZ3 should be viewed as a limitation of visualization, not a low-risk finding, and ECC appears most useful when combined with other markers of higher pretest risk.

Clinically, ECC is best interpreted as a triage tool rather than a stand-alone rule-out test. A positive ECC increases confidence in occult endocervical disease, whereas a negative or non-diagnostic ECC should be interpreted cautiously when high-risk features persist. In such cases, especially with ongoing discordance between screening risk and histology, a lower threshold for diagnostic excision may be appropriate.

## Figures and Tables

**Table 1:** Baseline characteristics of the TZ3 cohort by HPV group.

Variable	Total (*N* = 690)	HPV16/18 (*n* = 310)	Non-16/18 hrHPV (*n* = 380)	*p*-Value
Age, years (mean ± SD)	39.9 ± 9.4	38.9 ± 8.8	40.6 ± 9.8	0.019
Age ≥ 40 years	308 (44.6%)	121 (39.0%)	187 (49.2%)	0.009
High-grade cytology (ASC-H/HSIL)	214 (31.0%)	132 (42.6%)	82 (21.6%)	<0.001
High-grade colposcopic impression	182 (26.4%)	110 (35.5%)	72 (18.9%)	<0.001
ECC specimen insufficient	158 (22.9%)	66 (21.3%)	92 (24.2%)	0.414

Values are *n* (%) unless otherwise stated. *p*-values from Welch’s *t*-test (age) and chi-square tests (categorical variables). High-grade cytology includes ASC-H/HSIL. “Insufficient ECC” indicates inadequate material for histologic interpretation.

**Table 2:** Index composite histology and CIN2+ detection by biopsy and ECC (TZ3 cohort).

	Outcome	Total (*N* = 690)	HPV16/18 (*n* = 310)	Non-16/18 hrHPV (*n* = 380)	*p*-Value
Final histology	No dysplasia/CIN1	359 (52.0%)	122 (39.4%)	237 (62.4%)	<0.001
	CIN2	122 (17.7%)	60 (19.4%)	62 (16.3%)	0.298
	CIN3	198 (28.7%)	122 (39.4%)	76 (20.0%)	<0.001
	CIS	11 (1.6%)	6 (1.9%)	5 (1.3%)	0.555
	CIN2+ (CIN2/CIN3/CIS)	331 (48.0%)	188 (60.6%)	143 (37.6%)	<0.001
Detection pathway for CIN2+	Both biopsy+ECC CIN2+	97 (14.1%)	57 (18.4%)	40 (10.5%)	0.003
	Biopsy-only CIN2+	215 (31.2%)	117 (37.7%)	98 (25.8%)	0.001
	ECC-only CIN2+ (incremental yield)	19 (2.8%)	14 (4.5%)	5 (1.3%)	0.017
	Number needed to ECC to detect 1 additional CIN2+	36.3	22.1	76.0	—

CIN2+ includes CIN2, CIN3, and CIS. CIN3+ includes CIN3 and CIS. “ECC-only CIN2+” indicates CIN2+ detected on ECC with biopsy < CIN2. *p*-values for categorical comparisons were derived from chi-square tests or Fisher’s exact test, as appropriate; the number needed for ECC is a descriptive measure.

**Table 3:** Management and excisional outcomes among women with index histology below CIN2. (**A**) Indication for conization among women with index histology below CIN2 (*n* = 54); (**B**) Final excisional histology among women with index histology below CIN2 who underwent conization (*n* = 54).

**Variable**	***n*/*N***	**% of Respective *N***
Women with index histology below CIN2	359/690	52.0
Underwent subsequent conization within 12 weeks	54/359	15.0
Did not undergo conization	305/359	85.0
(**A**)		
**Variable**	***n*/*N***	**% of respective *N***
High-grade discordance in TZ3 despite index histology below CIN2	37/54	68.5
Insufficient ECC with persistent high-risk referral features	17/54	31.5
(**B**)		
**Variable**	***n*/*N***	**% of respective *N***
No dysplasia/CIN1	37/54	68.5
CIN2	6/54	11.1
CIN3	10/54	18.5
CIS	1/54	1.9
Upgraded to CIN2+ on conization	17/54	31.5

CIN2+ includes CIN2, CIN3, and carcinoma in situ (CIS). Percentages in the first three rows are calculated using the subgroup with index histology below CIN2 as denominator (*n* = 359). Percentages in the indication and excisional histology sections are calculated using the subgroup that underwent conization despite index histology below CIN2 as denominator (*n* = 54). TZ3, type 3 transformation zone; ECC, endocervical curettage.

## Data Availability

The original contributions presented in this study are included in the article. Further inquiries can be directed to the corresponding author.

## Abbreviations

CIS	carcinoma in situ
aOR	adjusted odds ratio
ASC-H	atypical squamous cells-cannot exclude HSIL
ASC-US	atypical squamous cells of undetermined significance
ASCCP	American Society for Colposcopy and Cervical Pathology
CI	confidence interval
CIN	cervical intraepithelial neoplasia
CIN2+	cervical intraepithelial neoplasia grade 2 or worse (CIN2, CIN3, or carcinoma in situ)
CIN3+	cervical intraepithelial neoplasia grade 3 or worse (CIN3 or carcinoma in situ)
ECC	endocervical curettage
EU	European Union
GDPR	General Data Protection Regulation
GLOBOCAN	Global Cancer Observatory cancer incidence and mortality database
HPV	human papillomavirus
hrHPV	high-risk human papillomavirus
HSIL	high-grade squamous intraepithelial lesion
LSIL	low-grade squamous intraepithelial lesion
NILM	negative for intraepithelial lesion or malignancy
R	R statistical computing environment
SCJ	squamocolumnar junction
SD	standard deviation
SPSS	Statistical Package for the Social Sciences
STROBE	Strengthening the Reporting of Observational Studies in Epidemiology
TZ	transformation zone
TZ3	type 3 transformation zone
WHO	World Health Organization
